# MicroRNA miR-27a-3p accelerates cardiac hypertrophy by targeting neuro-oncological ventral antigen 1

**DOI:** 10.1080/21655979.2022.2054150

**Published:** 2022-03-29

**Authors:** Dongyun Li, Mingzhi Shen, Xinxin Deng, Yongyi Bai

**Affiliations:** a Department of Healthcare, The Second Medical Center of Chinese PLA General Hospital, Beijing, China; b Department of Cardiovascular Medicine, Chinese Pla General Hospital Hainan Hospital, 80 Jianglin Road, Haitang District, Sanya, China; c Department of Inspection, Chinese Pla General Hospital Hainan Hospital, Sanya, China; d Department of Cardiovascular Medicine, The Second Medical Center of Chinese PLA General Hospital, Beijing, China

**Keywords:** Cardiac hypertrophy, miR-27a-3p, NOVA1

## Abstract

MiRNAs are a class of small non-coding RNAs (ncRNAs) responsible for post-transcriptional regulation of target genes. Accumulating evidence indicates that miRNAs are implicated in the progression of cardiac hypertrophy. Therefore, understanding the molecular mechanisms how these miRNAs regulate cardiac hypertrophy is useful for diagnosis and monitoring of disease progression. In this study, to investigate the effect of miR-27a-3p, we established an *in vitro* cardiac hypertrophy model by treating H9c2 cardiomyocytes with angiotensin II (Ang II) and an *in vivo* model through the chronic infusion of Ang II into mice. As revealed by our experimental results, miR-27a-3p expression was significantly increased in clinical samples, animal and cell models of cardiac hypertrophy. Inhibiting miR-27a-3p mitigated cardiac hypertrophy phenotype induced by Ang II. Additionally, our work identified NOVA1 (neuro-oncological ventral antigen 1) as a downstream target of miR-27a-3p. miR-27a-3p overexpression reduced NOVA1 protein level and mRNA expression. Consistently, NOVA1 silencing promoted cardiac hypertrophy phenotype induced by Ang II. In summary, these results suggest that the upregulation of miR-27a-3p may serve as a diagnostic factor for cardiac hypertrophy, and miR-27a-3p upregulation promotes cardiac hypertrophy by targeting NOVA1.

## Graphical abstract

**Figure:**
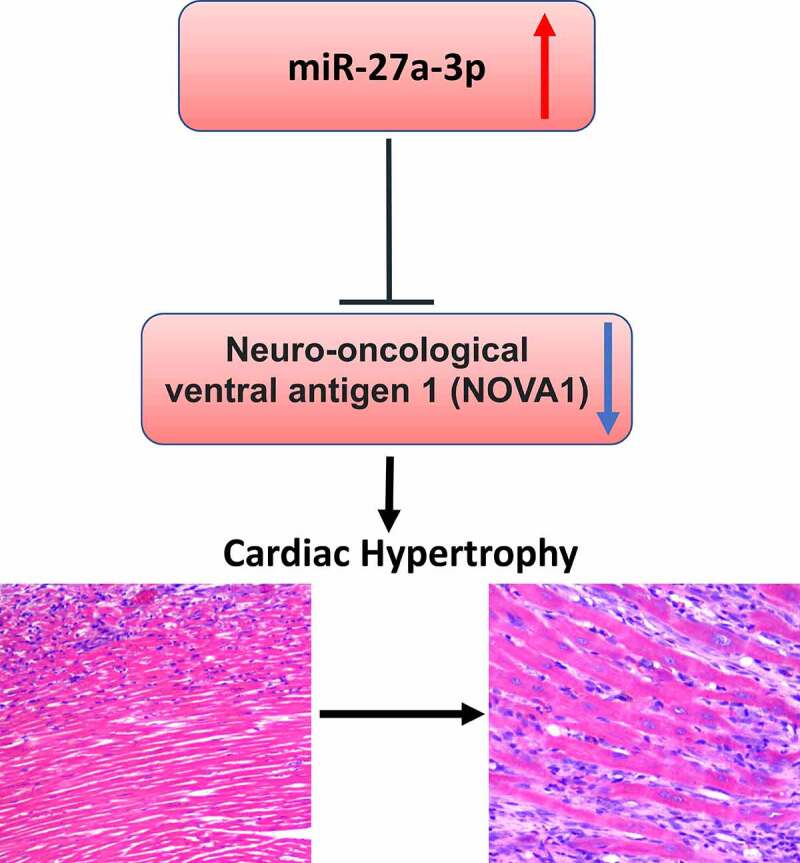

## Introduction

Cardiac hypertrophy is an adaptive response to hemodynamic stress, which is characterized by cardiomyocyte enlargement with no increase in cell quantity [1,2]. Cardiac hypertrophy is thought to function as a compensatory mechanism to reduce the workload pressure on ventricle walls, because the muscle fibers increase in size in ventricle walls [3]. Cardiac hypertrophy can be divided into pathological or physiological subtypes. Physiological hypertrophy is considered as mild and reversible changes, which frequently occurs in athletic activities, pregnancy, and infant growth [4,5]. In contrast, pathological hypertrophy results from chronic stresses such as diabetes, obesity, myocardial infarction (MI), and hypertension [6], and is characterized by an excessive increase in ventricular size, and myocardial dysfunction and fibrosis [7,8]. When left untreated, pathological hypertrophy could eventually result in heart failure (HF) or even death.

MicroRNAs (miRNAs), with a length of 18–22 nucleotides, are evolutionarily conserved non-coding RNAs [9–11]. They are important regulators of various intracellular signaling pathways and play critical roles in regulating gene expression [12]. It has been found that cardiovascular disease (CVD)-related genes can be regulated through the interaction of miRNAs with 3'-untranslated region (UTR) [13–15]. The deregulation of microRNAs has been reported to promote cardiac hypertrophy, through modulating genes involved in hypertrophic signaling pathways [16,17], such as miR-22, miR-29, miR-155, miR-217, and miR-200c [18–22]. A comprehensive picture of miRNAs dysregulated in cardiac hypertrophy will be useful to understand gene regulatory network underlying cardiac hypertrophy development.

MiR-27a-3p has been identified as an oncogene that is frequently dysregulated in various human cancers [23–26]. In addition, miR-27a-3p has been reported to be implicated in CVD development, including myocardial infarction [27]. Recently, miR-27a-3p is found to be dysregulated in hypoxia-induced cardiomyocyte injury [27]. However, its expression pattern and functional role in cardiac hypertrophy are still unclear.

NOVA1 (neuro-oncological ventral antigen 1) is an RNA-binding protein that was first characterized in neurons [28,29]. NOVA1 plays a critical role in cancer initiation and progression [30–32]. Li, et al. reported that NOVA1 is a downstream target of miR-27a-3p and could promote epithelial mesenchymal transition (EMT) in gastric cancer [33]. However, its function in cardiac hypertrophy remains to be characterized.

In this study, we established a mouse model of cardiac hypertrophy by the infusion of Ang II and an *in vitro* cell model based on H9c2 cells. We aimed to investigate the expression pattern and functional role of miR-27a-3p in Ang II-induced cardiac hypertrophy, identify the downstream target of miR-27a-3p, and determine whether the miR-27a-3p/NOVA1 axis regulates the progression of Ang II-induced cardiac hypertrophy. According to our results, miR-27a-3p expression was upregulated in cardiac hypertrophy. We further demonstrated that miR-27a-3p promoted cardiac hypertrophy through regulating NOVA1 expression.

## Materials and methods

### Clinical samples

Altogether a total number of 30 cardiac hypertrophy cases were enrolled in the present work at The Second Medical Center of Chinese PLA General Hospital between January 2019 and December 2020. The 30 patients were diagnosed with tetralogy of Fallot or trilogy of Fallot by echocardiography, and with an age span of 38–76 years (average age of 53.5 years), as well as with clinical sign of cardiac hypertrophy. Of the 30 patients, 16 were males and 14 were females. The clinical features of the patents are summarized in Table 1.

**Table 1.** Summary of baseline clinical features, laboratory results and imaging characteristics of study sample

Variable	Control (n = 30)	HCM (n = 30)	*P* Value
Age (years)	56.1 ± 1.4	54.8 ± 1.5	0.550
Male (Female)	15 (15)	17 (13)	N/A
Family history of HCM, n (%)	0	2 (6)	N/A
Body mass index	20.3 ± 0.3	21.2 ± 0.4	0.091
NYHA class (I/II/III/IV)	0/0/0/0	9/21/0/0	< 0.001
**Drugs**			
ACE inhibitors	0	6 (20)	N/A
Antiarrhythmic agents	0	1 (3)	N/A
Diuretics, n (%)	0	3 (10)	N/A
Aldosterone, n (%)	0	5 (17)	N/A
β-blockers, n (%)	0	17 (57)	N/A
Calcium channel blockers, n (%)	0	10 (33)	N/A
Aspirin, n (%)	0	12 (40)	N/A
**Comorbidities**			
Hypertension, n (%)	0	0 (0)	N/A
Diabetes mellitus, n (%)	0	3 (10)	N/A
Chronic kidney disease, n (%)	0	1(3)	N/A
COPD	0	1(3)	N/A
**Echocardiographic features**			
Left ventricular ejection fraction (%)	68.7 ± 1.2	69.1 ± 1.2	0.814
Intraventricular septal thickness (mm)	9.3 ± 0.2	17.4 ± 0.5	< 0.001
Left ventricular posterior wall thickness (mm)	8.9 ± 0.2	18.1 ± 0.6	< 0.001
Left ventricular outflow tract gradient (mm Hg)	19.7 ± 0.7	84.6 ± 1.7	< 0.001
Plasma brain natriuretic peptide (100 pg/mL)	54.8 ± 2.2	558.4 ± 24.7	< 0.001
Heart rate (bpm)	71.3 ± 1.1	74.2 ± 1.2	0.083
Systolic blood pressure (mmHg)	122.5 ± 1.1	119.8 ± 1.2	0.092
Diastolic blood pressure (mmHg)	70.8 ± 1.3	69.4 ± 1.2	0.418

Mean ± SEM; N/A = Not Available

The peripheral blood samples were collected from the patients and healthy control subjects. Serum was prepared and stored at −80°C until usage. The study protocols were approved by the Medical Ethics Committee of the Second Medical Center of Chinese PLA General Hospital. The procedures involving human participants in this study were in line with the guidelines of Declaration of Helsinki. All participants signed the informed consent.

### Animal model

A total number of 12 Eight-week-old male C57BL/6 mice weighing 20 ± 2 g were maintained under a specific pathogen-free condition with free access to tap water and regular mice chow pellet. The mice were randomly assigned to Ang II infusion and sham group (n = 6 in each group). A cardiac hypertrophic condition was induced by Ang II infusion (Ang II dissolved in PBS with 10 µmol/L acetic acid) at a dose of 2.5 µg/kg/minute with a subcutaneously implanted minipump (model 2002, Alza, Moun tain View, CA, USA) for 15 days [34]. Mice with PBS infusion served as the sham group. All the animal procedures followed the Guide for the Care and Use of Laboratory Animals (US and National Institutes of Health). The animals from each group were euthanized in a CO_2_ chamber after treatment. Afterward, ventricles were dissected and used for HE staining and western blot to check the marker of cardiac hypertrophy. The animal study protocols were approved by the Medical Ethics Committee of the Second Medical Center of Chinese PLA General Hospital.

### Cell culture and treatment

Rat heart-derived H9c2 cells were cultured using DMEM with 10% fetal bovine serum (FBS) and 100IU/mL of penicillin–streptomycin under 37°C and 5% CO_2_ conditions. H9c2 cells were subjected to 48 h treatment of 1.0 mmol/L Ang II or PBS [34]. NOVA1-siRNA, miR-27a-3p inhibitor, miR-27a-3p mimic and scramble negative controls were purchased from RiboBio Co., Ltd. (Guangzhou, China). Cell transfection was performed with 100 nM NOVA1-siRNA, 100 nM miR-150 mimic or inhibitor using the Lipofectamine 2000 (Invitrogen, Thermo Fisher Scientific, Inc., Waltham, MA, USA) based on the manufacturer’s instructions. Experiments were performed 48 h post-transfection.

### RNA extraction and RT-qPCR

Total blood and cellular RNAs were isolated using Trizol reagent (Invitrogen) according to manufacturer’s protocol. The first strand cDNA was synthesized using a First-Strand Synthesis kit (Takara, Dalian, China), and RT-qPCR was performed using gene-specific primers and SYBR premix EX TAQ II kit (RR820A, Takara, Dalian, China), as described previously **[**34**]**. The following PCR conditions were used: denaturation at 95°C for 10 min and 45 amplification cycles (denaturation at 95°C for 15 s, annealing and extension at 60°C for 1 min). RT-qPCR was performed in a 7500 Real-Time PCR System (Applied Biosystems, Carlsbad, CA, USA). The 2–∆∆Ct method was used to analyze the relative expression level and GAPDH was used as the internal reference gene.

RT-qPCR primers used in this study: miR-27a-3p-F: 5'-CCGCTCGAGACTGGCTGCTAGGAAGGTG-3' and miR-27a-3p-R: 5'-GCGAATTCTTGCTGTAGCCTCCTTGTC-3'.

GAPDH-F: 5'-AGCCAAAAGGGTCATCATCT-3' and GAPDH-R: 5'GGGGCCATCCACAGTCTTCT-3'.

NOVA-1-F: 5'-TCAGCAGAACGGGACCCATA-3' and NOVA-1-R: 5'-GGGGTCAGATGGAGAGGACT-3'

ANP-F: 5'-ACCTGCTAGACCACCTGGAG-3' and ANP-R: 5'-CCTTGGCTGTTATCTTCGGTACCGG-3'

β-MHC-F: 5'-CGCTACGCTTCCTGGATGAT-3'and β-MHC-R: 5'-TCCGGTGATGAGGATGGACT-3'

BNP-F: 5'-CGCTGGGAGGTCACTCCTAT-3' and BNP-R: 5'-CTCCAGCAGCTTCTGCATCT-3'

### Cell surface area determination

After treatment, cells were fixed by 4% paraformaldehyde and permeabilized with 0.1% Triton X-100 in PBS. After blocking using 5% BSA, the cells were incubated with anti-α-actinin at a dilution of 1:100 in 1% goat serum overnight at 4°C, and then incubated with Alexa Fluor ® 594 goat anti-mouse IgG for 1 h at 37°C. Cells were imaged using a fluorescent microscope at 600x magnification (Olympus model, Tokyo, Japan) and Image J software was adopted for examining cell surface area by setting a threshold for measuring α-actinin staining signals. 100 cells were randomly selected to measure surface area in three wells of independent treatment [35].

### Western blotting (WB) analysis

Total protein was extracted from tissues or cells using RIPA lysis buffer containing protease inhibitor cocktail (Thermo Fisher Scientific 78,429, Waltham, MA, USA). Lysed cells were centrifuged at 14,000 rpm for 10 mins and the protein concertation in the supernatant was quantified by a BCA Protein assay kit (Beyotime, Shanghai, China). A total of 20 ug protein was used for SDS-PAGE electrophoresis and transferred onto the PVDF membrane (BioRad, Irvine, CA, USA). After blocking with 5% skimmed milk for 1 hour, the membrane was then incubated with primary antibodies: anti-NOVA1 (ab183024, rabbit, 1:1000), anti-ANP (PA5-29,559, rabbit, 1:2000), anti-β-MHC (ab170867, rabbit, 1:1000), anti-BNP (ab19645, rabbit, 1:1000) and anti-GAPDH (ab8245, mouse, 1:2000) antibodies (Abcam, Cambridge, MA, USA) overnight 4°C. After that, the membranes were washed with TBST buffer and further incubated at room temperature with Goat Anti-Rabbit IgG H&L (HRP) (ab6721, 1:2500), or Goat Anti-Mouse IgG H&L (HRP) (ab205719, 1:2500) (Abcam, Cambridge, MA, USA) for 90 minutes. The specific protein bands were developed using an enhanced chemiluminescence kit (Santa Cruz, TX, USA, sc-2048) and photographed on a gel imager system (Bio-Rad, Hercules, CA, United States) [35]. GADPH served as the loading control.

### Dual Luciferase reporter assay

To demonstrate the functional interaction between miR-27a-3p and NOVA1 mRNA, the sequence containing the wild-type binding site in 3'UTR of NOVA1 mRNA or the sequence with mutated binding site were cloned into the PmirGLO firefly luciferase vector (Promega, CA, USA). The reporter plasmid and Renilla luciferase (hRlucneo) control plasmid were co-transfected into cells in the presence of miR-27a-3p mimic or miR-NC using Lipofectamine 2000 reagent. 48 h after transfection the relative luciferase activities were measured using Dual-Luciferase Reporter Assay Kit (Promega, CA, USA) on a microplate reader.

### Histopathologic analysis

Hematoxylin and Eosin (H&E) Staining was performed using H&E Stain Kit (ab245880, Abcam). Deparaffinized/hydrated sections of mouse ventricles were incubated in adequate Hematoxylin, Mayer’s (Lillie’s Modification) to completely cover tissue section for 5 mins. The section was rinsed twice with distilled water and incubated with adequate Bluing Reagent for 30 secs. After washing with distilled water, the section was dehydrated in absolute alcohol, followed by staining with Eosin Y Solution to completely cover tissue for 2–3 mins. The section was rinsed using absolute ethanol for three times and then mounted to a slide, and the images were collected under an inverse microscope [35].

### Statistical analysis

GraphPad Prism 8.0 Software (GraphPad Software, Inc.) was utilized for statistical analysis. The statistical difference between two groups was compared using unpaired Student’s *t* test. Multiple group comparisons were detected using analysis of variance (ANOVA) with Tukey’s post hoc test for pairwise comparison. All the experiments were repeated three times. The significance level was set at P < 0.05.

## Results

In this study, to investigate the expression and functional role of miR-27a-3p in cardiac hypertrophy, we established a mouse model of cardiac hypertrophy by the infusion of Ang II and an *in vitro* cell model based on H9c2 cells. Based on our results, miR-27a-3p expression was upregulated in the animal and cell model of cardiac hypertrophy. We further demonstrated that miR-27a-3p promoted cardiac hypertrophy through regulating NOVA1 expression.

### MiR-27a-3p expression is upregulated in peripheral blood samples of cardiac hypertrophy patients

**Figure 1.**
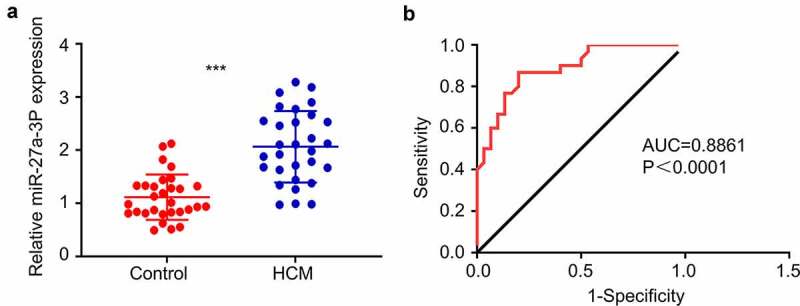
**MiR-27a-3p is upregulated in peripheral blood samples of patients with cardiac hypertrophy**. (a) RT-qPCR analysis of miR-27a-3p level in the peripheral blood samples of 30 patients with cardiac hypertrophy and 30 healthy controls. GAPDH was used as the housekeeping gene for normalization in all the following experiment. (b) AUC curve analysis of the predictability of miR-27a-3p in diagnosis of cardiac hypertrophy in blood samples. ***P < 0.001.

We first performed RT-qPCR analysis for miR-27a-3p expression level in the peripheral blood samples of 30 cardiac hypertrophy patients and 30 healthy controls. The patients showed different degrees of heart malfunction according to New York Heart Association (NYHA) Functional Classification, as well as clinical signs of cardiac hypertrophy detected by Echocardiography (Table 1). miR-27a-3p expression levels were significantly elevated in blood samples of cardiac hypertrophy patients as compared to healthy controls (Figure 1a). ROC curve analysis showed that the area under ROC curve (AUC) is greater than 0.8, indicating that miR-27a-3p level could serve as good indicator for the diagnosis of cardiac hypertrophy (Figure 1b). These results suggest that miR-27a-3p upregulation in peripheral blood could be used as a potential diagnostic factor for cardiac hypertrophy.

### MiR-27a-3p upregulation in mouse model of angii-induced cardiac hypertrophy

**Figure 2.**
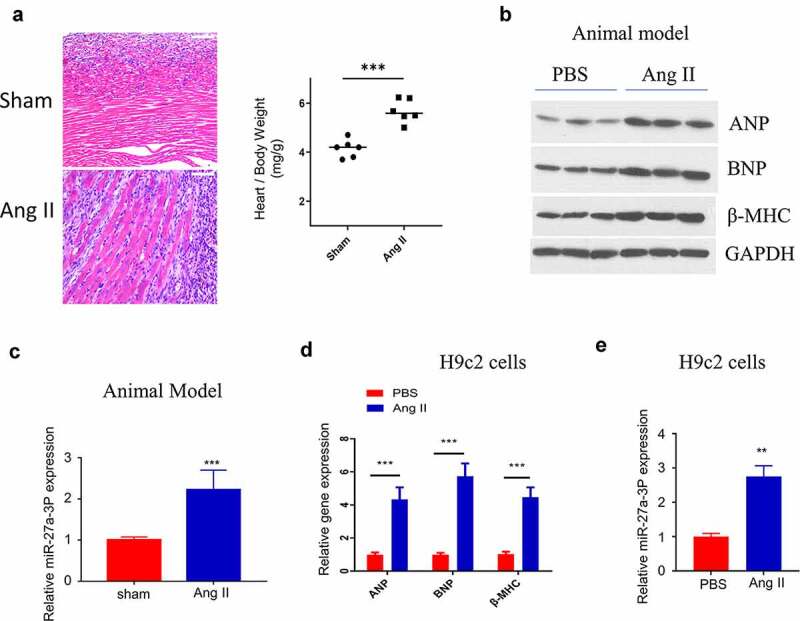
**MiR-27a-3p is upregulated in Ang II–induced cardiac hypertrophy model**. (a) H&E staining of the ventricular sections in sham and Ang II–infused mouse. Scale bar: 200 µm. The measurement of heart weight/body weight was shown in the right panel (n = 6 mice in each group). (b) Western blot analysis of hypertrophic markers: atrial natriuretic peptide (ANP), B-type natriuretic peptide (BNP), β-myosin heavy polypeptide (MHC) in ventricles samples of sham and Ang-II treated mouse. The results of 3 independent samples of each condition were analyzed. (c) MiR-27a-3p expression analysis by RT-qPCR in blood sample of mouse with sham or Ang II treatment (n = 6 mice in each group). (d) qRT-PCR analysis of the expression of hypertrophic markers: atrial natriuretic peptide (ANP), B-type natriuretic peptide (BNP),β-myosin heavy polypeptide (MHC) in H9c2 cells after Ang II treatment. (e) miR-27a-3p expression in H9c2 cells after Ang II treatment for 48 h. n = 3 independent experiments in (D) and (E). **P < 0.01, ***P < 0.001.

We next examined miR-27a-3p level in a mouse model of AngII–induced hypertrophy. Ang II–infusion caused significant sign of cardiac hypertrophy, as revealed by the enlargement of the cardiac fiber by H&E staining, as well as the increased heart weight (Figure 2a). We examined the expression of hypertrophic marker genes, including ANP (Atrial Natriuretic Peptide), BNP (Natriuretic Peptide B) and β-MHC (β-Myosin Heavy Chain) [36]. The results showed that the expression of ANP, BNP and β-MHC was significantly upregulated in ventricle samples of Ang II–infused mice, indicating the induction of cardiac hypertrophy by Ang II infusion (Figure 2b). Consistently, miR-27a-3p level within peripheral blood of mice after 15 days of Ang II–infusion significantly increased relative to sham control (Figure 2c). Moreover, we also established an *in vitro* cell model by treating H9c2 cells with Ang II. ANP, BNP and β-MHC expression were significantly upregulated at both protein and mRNA levels in H9c2 cells after Ang II treatment (Figure 2d). In this cell model, we also confirmed that Ang II treatment induced a significant increase of miR-27a-3p expression (Figure 2e).

### Inhibiting MiR-27a-3p Attenuates Cardiac Hypertrophy caused by Ang II

**Figure 3.**
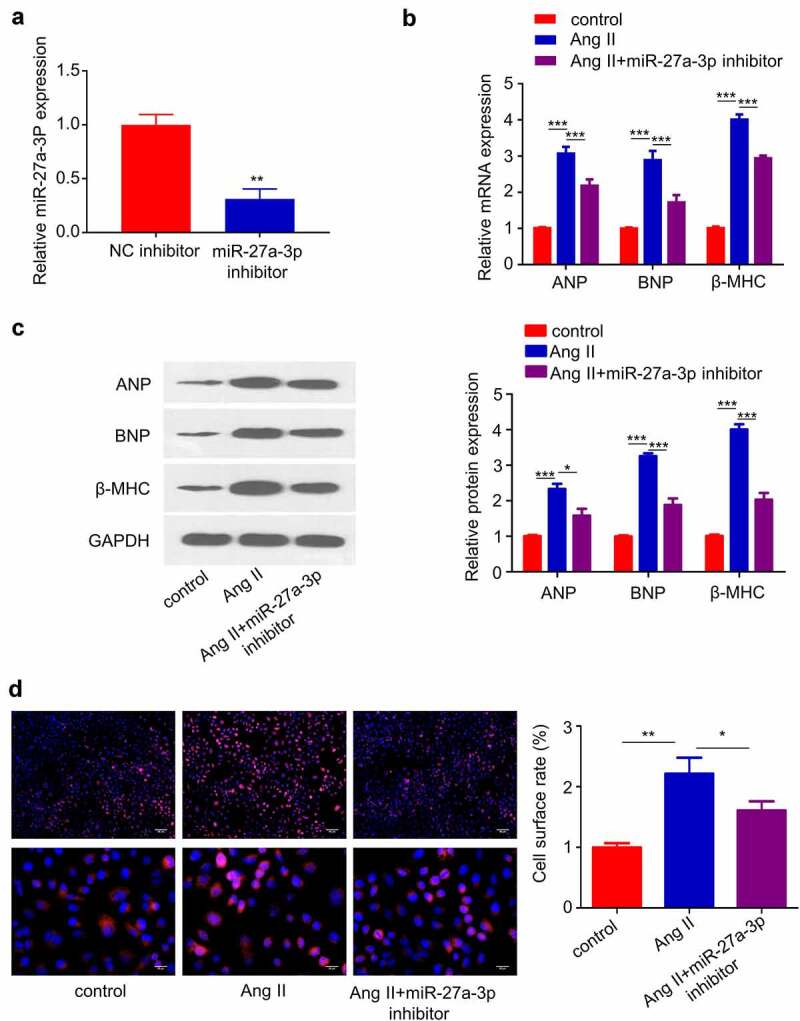
**miR-27a-3p inhibition suppresses Ang II–induced cardiac hypertrophy**. (a) miR-27a-3p expression level in H9c2 cells after the transfection of miR-27a-3p inhibitor. n = 3 independent experiments. (b), β-MHC and BNP mRNA levels were detected using RT-qPCR in H9c2 cells. n = 3 independent experiments. (c) Western blotting analysis of the protein levels of, β-MHC and BNP in H9c2 cells with indicated treatment. Relative level of the target proteins was normalized to GAPDH level. n = 3 independent experiments. (d) α-actinin immunofluorescence staining and cell surface area determination. Scale bar: 20 µm. n = 3 independent experiments., each with the measurement of 100 cells. *P < 0.05, **P < 0.01, ***P < 0.001.

We next sought to validate the role of miR-27a-3p in Cardiac Hypertrophy. We transfected miR-27a-3p inhibitor into H9c2 cells and RT-qPCR analysis confirmed that the transfection of inhibitor suppressed miR-27a-3p expression (Figure 3a). In addition, we assessed whether miR-27a-3p inhibition attenuated the expression of cardiac hypertrophy marker genes. RT-qPCR analysis showed that the upregulation of, BNP and β-MHC induced by Ang II was significantly suppressed by miR-27a-3p inhibition (Figure 3b, c). Furthermore, H9c2 cell surface area was determined by α-actinin staining, and the results showed that Ang II treatment for 48 h substantially increased cell surface area, which was inhibited by miR-27a-3p inhibitor (Figure 3d). These data suggest that miR-27a-3p upregulation contributed to cardiac hypertrophy in H9c2 cells.

### NOVA1 is a target of miR-27a-3p in cardiac hypertrophy

**Figure 4.**
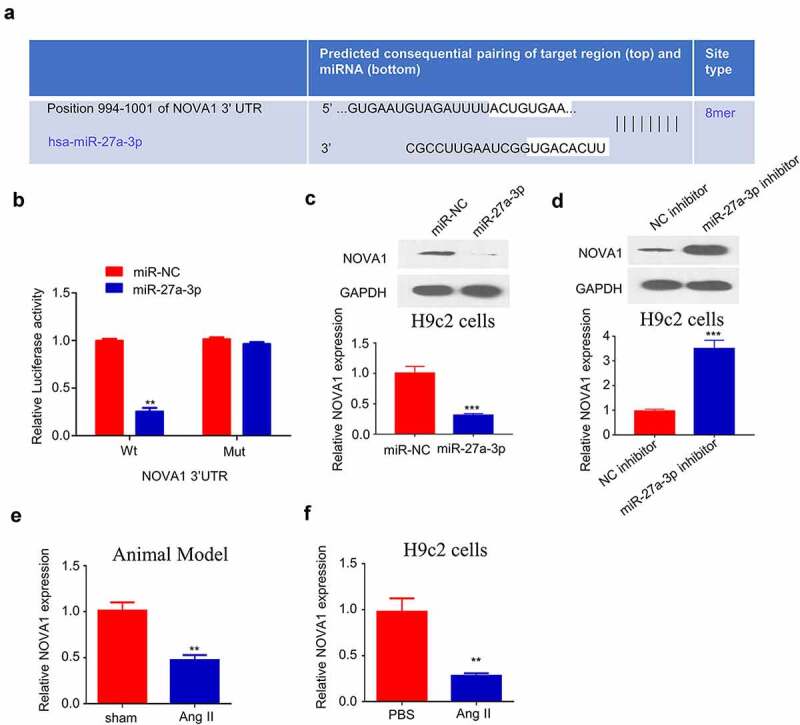
**NOVA1 is a target negatively regulated by miR-27a-3p**. (a) Bioinformatics analysis on binding site between NOVA1 mRNA 3'UTR and miR-27a-3p. n = 3 independent experiments. (b) Dual luciferase reporter assay using WT-NOVA1 3'UTR reporter and Mut reporter, in the presence of miR-27a-3p mimic or miR-NC. n = 3 independent experiments. (c) NOVA1 level in H9c2 cells transfected with miR-27a-3p mimic was measured through RT-qPCR and WB assay. n = 3 independent experiments. (d) NOVA1 level in H9c2 cells transfected with miR-27a-3p inhibitor was measured through RT-qPCR and WB assays. Protein band density of the target proteins were normalized to GAPDH and expressed as fold change. n = 3 independent experiments. (e,f) NOVA1 mRNA levels in the blood samples of Ang II infusion mice and Ang II-treated H9c2 cells were measured by RT-qPCR. n = 3 independent experiments. **P < 0.01, ***P < 0.001.

Since miR-27a-3p showed an important role in cardiac hypertrophy, we further used TargetScan (http://www.targetscan.org/) to predict the downstream target mRNAs of miR-27a-3p. Based on the prediction, binding site between the 3’-UTR of NOVA1 mRNA and miR-27a-3p sequence was shown at 994–1001 (Figure 4a). We next performed dual-luciferase reporter assay to confirm their interaction. The results showed that the activity of NOVA1 3'UTR-WT reporter was significantly inhibited by miR-27a-3p mimic, while no effect was observed when the binding sequence was mutated (Figure 4b). These data hinted that miR-27a-3p specifically bind to the 3'-UTR of NOVA1 mRNA. Furthermore, we performed RT-qPCR and Western blot to investigate how miR-27a-3p overexpression or inhibition regulates NOVA1 mRNA and protein expression. The transfection of miR-27a-3p mimic remarkably reduced NOVA1 protein and mRNA levels (Figure 4c), while miR-27a-3p inhibitor increased NOVA1 expression (Figure 4d). Meanwhile, we examined NOVA1 levels in the ventricular tissues of animal model as well as in cells treated with Ang II. We found that NOVA1 level in the ventricular tissues of Ang II-infused mice and Ang II-treated H9c2 cells showed a significant decrease compared with controls (Figure 4e, F). Therefore, NOVA1 is a target of miR-27a-3p involved in cardiac hypertrophy regulation.

### MiR-27a-3p enhances cardiac hypertrophy partially through targeting NOVA1

**Figure 5.**
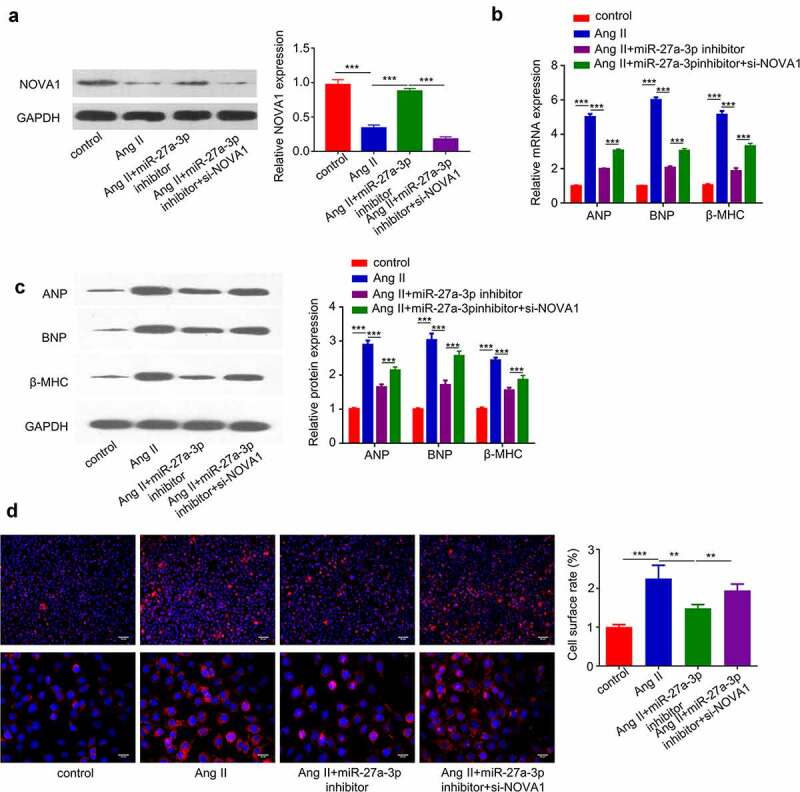
**MiR-27a-3p enhances cardiac hypertrophy partially through targeting NOVA1**. (a). Ang II-treated H9c2 cells were transfected with miR-27a-3p inhibitor or miR-27a-3p inhibitor +NOVA1 siRNA. Western blot was performed to examine NOVA1 protein levels. n = 3 independent experiments. (b) RT-qPCR analysis of mRNA levels of, β-MHC and BNP in H9c2 cells with above treatment. n = 3 independent experiments. (c) Western blot analysis of, β-MHC and BNP in H9c2 cells with above treatment. n = 3 independent experiments. For Western blot, protein bands of target proteins were normalized to GAPDH and expressed as fold change. (d) Cell surface area analysis in H9c2 cells with above treatment. n = 3 independent experiments, each with the measurement of 100 cells. **P < 0.01, ***P < 0.001.

To further corroborate the role of miR-27a-3p/NOVA1 axis in cardiac hypertrophy, we treated H9c2 cells with Ang II, in the presence of miR-27a-3p inhibitor and NOVA1 siRNA. Western blot analysis revealed that Ang II reduced NOVA1 protein level, which was partially rescued by miR-27a-3p inhibitor and NOVA1 siRNA abrogated the rescue effect (Figure 5a). We then examined the expression of hypertrophic markers (β-MHCand BNP) at protein and mRNA level. The induction of hypertrophic markers by Ang II was largely suppressed by miR-27a-3p inhibitor, and NOVA1 silencing partially counteracted the suppression effect (Figure 5b, c). Similarly, silencing NOVA1 partially abrogated the rescue effect of miR-27a-3p inhibitor on cell surface enlargement (Figure 5d). These results altogether support that miR-27a-3p/NOVA1 axis mediates cardiac hypertrophy in H9c2 cells.

## Discussion

Cardiac hypertrophy is a common response to chronic and acute cardiac injury, which could eventually leads to ventricular hypertrophy, dilation, and heart failure [37]. In the past years, accumulating evidence indicates that miRNAs contributes to the pathological progression of cardiac hypertrophy. miR-208a overexpression has been reported to cause the upregulation of hypertrophy gene and increase the cell size [38]. Another study reported that aberrant expression of miR-29a can promote cardiac hypertrophy through targeting hypertrophic signaling pathways [16,17]. These results indicate that the deregulation of miRNAs plays critical roles in cardiac hypertrophy progression.

In this study, we examined the expression and role of miR-27-3p in clinical samples, animal model and cell model of cardiac hypertrophy. miR-27a-3p expression is remarkably increased in clinical peripheral blood from cardiac hypertrophy patients, as well as in the animal model and cell model. Inhibition of miR-27a-3p can reduce Ang II-induced upregulation of hypertrophic markers (β-MHC and BNP), and also alleviates the increase of cell surface area in H9c2 cells. These data suggest the potential contribution miR-27-3p to the progression of cardiac hypertrophy. These findings were consistent with the reported role of miR-27a-3p in other cardiovascular disorder [27].

We further demonstrated that NOVA1 is a potential miR-27a-3p target gene. In this study, we found that there was a binding site for miR-27a-3p at the 3’-UTR of NOVA1. Although a previous study has shown that NOVA1 is implicated in gastric cancer (GC) progression via interacting with miR-27a-3p [33], there is no report regarding the role of NOVA1 on cardiac hypertrophy. We confirmed their interaction by dual-luciferase reporter assay and demonstrated that NOVA1 expression level could be negatively regulated by miR-27a-3p expression. In addition, in both animal model and cell model of cardiac hypertrophy, we showed the reduced expression of NOVA1. These results suggested novel functions of NOVA1 in regulating cardiovascular cells.

Previous studies mainly focused on the roles of miR-27a-3p in cancer progression. MiR-27a-3p upregulation has been identified in a variety of human cancers, and miR-27a-3p modulates cancer progression by targeting multiple targets including NOVA1, FBXW7 and BTG2 [23,24,26]. MiR-27a-3p regulates the expression of NOVA1 to control epithelial-mesenchymal transition in gastric cancer [33]. Since NOVA1 is mainly investigated as a regulator in neuron cells [29], future work needs to further characterize the functional mechanisms by which NOVA1 regulates tumorigenesis and the function of cardiovascular cells.

## Conclusion

In summary, we reported that miR-27a-3p is upregulated in patients of cardiac hypertrophy and in Ang II-induced models of cardiac hypertrophy. Inhibiting miR-27a-3p alleviates cardiac hypertrophy phenotype induced by Ang II. Additionally, our work corroborated the contribution of miR-27a-3p/NOVA1 axis to cardiac hypertrophy phenotype. These results suggest that miR-27a-3p upregulation may serve as a diagnostic marker for cardiac hypertrophy, and miR-27a-3p upregulation promotes cardiac hypertrophy by targeting NOVA1.
